# Early results from the use of an innovative vertical fascial traction system for the management of patients with open abdomen

**DOI:** 10.3389/fsurg.2025.1644791

**Published:** 2025-11-11

**Authors:** Orestis Ioannidis, Aliki Brenta, Alexis Theodorou, Konstantinos Siozos, Georgios Gemousakakis, Εlissavet Anestiadou, Ekaterini Klonou, Savvas Konstantinos Symeonidis, Stefanos Bitsianis, Efstathios Kotidis, Ioannis Mantzoros, Manousos Georgios Pramateftakis, Stamatios Angelopoulos

**Affiliations:** 1 4th Department of Surgery, School of Medicine, Faculty of Health Science, General Hospital of Thessaloniki “G. Papanikolaou”, Aristotle University of Thessaloniki, Thessaloniki, Greece; 2 1st Propaedeutic Department of Surgery, University of Athens, Hippocratio Hospital Athens, Athens, Greece

**Keywords:** open abdomen, fascial traction, fascial closure device, trauma, sepsis

## Abstract

**Background:**

The combination of negative pressure wound therapy (NPWT) with dynamic fascial traction is currently considered the preferred method for temporary closure of the open abdomen (OA). However, this approach often requires repeated returns to the operating room for further fascial approximation. The aim of this study was to present our institution's experience with a vertical fascial traction device (VTD) for OA management and early closure.

**Methods:**

This is a prospective registry of patients treated with the VTD between May 2023 and the present. The system used is commercially named Fasciotens® Abdomen, manufactured by Fasciotens GmbH (Essen, Germany).

**Results:**

Definitive abdominal closure was achieved in 11 of 13 patients. Eight patients underwent primary midline suture, while 3 patients—all with pre-existing hernias—required mesh reinforcement. Two patients died before closure could be performed.

**Conclusions:**

The vertical fascial traction device applies continuous upward traction to the rectus abdominis fascia through an external frame anchored to the pelvis and thorax, thereby increasing abdominal compartment volume and reducing intra-abdominal pressure. This innovative technique facilitates earlier and safer abdominal wall closure and represents a promising adjunct in the management of the open abdomen.

## 1 Introduction

The open abdomen (OA) is, by definition, the intentional decision to leave the fascial edges unapproximated after laparotomy (1). This approach is an acceptable damage-control management strategy following damage-control surgery in severely injured or critically ill patients, as it facilitates resuscitation, subsequent re-exploration, and definitive control of abdominal pathology (1–3). OA management prevents intra-abdominal hypertension and abdominal compartment syndrome, provided that early fascial closure is ultimately achieved once source control is secured and no further surgical interventions are anticipated (2, 3). However, prolonged OA has been associated with increased complications, including bowel adhesions, entero-atmospheric fistulas, intra-abdominal abscesses, and complex abdominal wall hernias due to loss of domain (4, 5). For this reason, early primary fascial closure should be a principal goal in OA management (6).

Several techniques have been developed to facilitate fascial approximation after laparotomy, with the primary aim of achieving direct closure (7, 8), although biologic mesh reinforcement has also been used in selected cases (9–12). Notably favorable outcomes have been reported with the combination of vacuum therapy and fascial traction, either through mesh-mediated techniques such as vacuum-assisted wound closure and mesh-mediated fascial traction (VAWCM), or by using dynamic closure systems such as the ABRA® abdominal wall closure device (13).

The combination of negative pressure wound therapy (NPWT) with dynamic fascial traction has been shown to yield high rates of primary closure in OA patients (14, 15). In the setting of urgent laparotomy, intraoperative measures may include mesh-mediated horizontal traction or fascial traction. The latter can be achieved using a vertical fascial traction device (VTD), which applies controlled myofascial elongation and thereby enables direct fascial closure.

This study reports the successful introduction and clinical use of an innovative device designed to apply dynamic vertical traction to the abdominal fascia in a cohort of 13 patients treated over the past 20 months. Definitive abdominal closure was achieved in 11 of 13 patients, including 8 with primary midline suture and 3 with mesh reinforcement. The device employed is commercially available as the Fasciotens® Abdomen (Fasciotens GmbH, Essen, Germany), hereafter referred to as the vertical traction device (VTD) for brevity.

## 2 Materials and methods

This prospective observational study systematically registered and analyzed patients undergoing open abdomen (OA) management, focusing on demographic, clinical, and surgical factors. We examined patient demographics, including gender and age (median age 60.5 years), as well as the underlying etiology for OA, such as peritonitis, imminent abdominal compartment syndrome (ACS), intra-abdominal hemorrhage, and generalized ileus. Surgical risk was assessed using the ASA classification (III–V), and prior abdominal surgeries were recorded. Comorbidities,-including diabetes mellitus, myocardial disease, arterial hypertension, COPD, and renal insufficiency, were documented together with the Charlson Comorbidity Index and APACHE score and were evaluated for their potential impact on patient outcomes. Additionally, the need for ICU treatment prior to the first surgery was also evaluated, with attention to mechanical ventilation, antibiotic use, and vasopressor support.

Key surgical outcomes included mortality before fascial closure, achievement of definite fascial closure (DFC), the total number of surgical revisions, days to DFC, and fascia-to-fascia (FTF) distance at OA. Finally, the method of closure was analyzed, distinguishing between mesh-mediated closure and primary midline suture, both performed in combination with negative pressure wound therapy (NPWT), to evaluate their effectiveness in achieving abdominal closure.

### 2.1 Patient selection and indications for vertical traction device (VTD) use

During the study period (May 2023 to present), a total of 37 patients required open abdomen (OA) management at our institution. Of these, 13 consecutive patients fulfilled the predefined criteria for vertical fascial traction device application and were treated with the Fasciotens® Abdomen (Fasciotens GmbH, Essen, Germany). Patients were selected based on clinical indications necessitating temporary abdominal closure with fascial traction. Indications for OA included damage control surgery for peritonitis, intra-abdominal hemorrhage, abdominal compartment syndrome (ACS), and bowel obstruction with generalized ileus.

The decision to use the VTD was made by the attending surgical team when dynamic fascial traction combined with negative pressure wound therapy (NPWT) was considered necessary to facilitate early fascial closure. In accordance with damage control principles, the device was never applied at the index laparotomy in order to minimize operative time. Instead, inclusion criteria were assessed intraoperatively at the first planned revision laparotomy (typically the second surgical procedure). Specifically, the VTD was employed when it was determined that primary approximation of the fascial edges could not be achieved without traction assistance, and such closure was not anticipated to be feasible even at the subsequent planned re-exploration.

The explicit indications for VTD use were:

(i) damage control surgery for peritonitis with inability to achieve primary fascial closure,
(ii) intra-abdominal hemorrhage requiring OA management,
(iii) abdominal compartment syndrome (ACS), and
(iv) bowel obstruction with generalized ileus, where direct fascial approximation was not possible.

The predefined contraindications were:

(i) patient hemodynamic instability necessitating abbreviated surgical procedures, and
(ii) situations where fascial closure could be achieved primarily without traction assistance.

During the study period, no patients were excluded based on these contraindications, reflecting a consecutive enrollment approach that mirrors routine clinical practice.

### 2.2 Description of surgical technique

**Figure 1:**
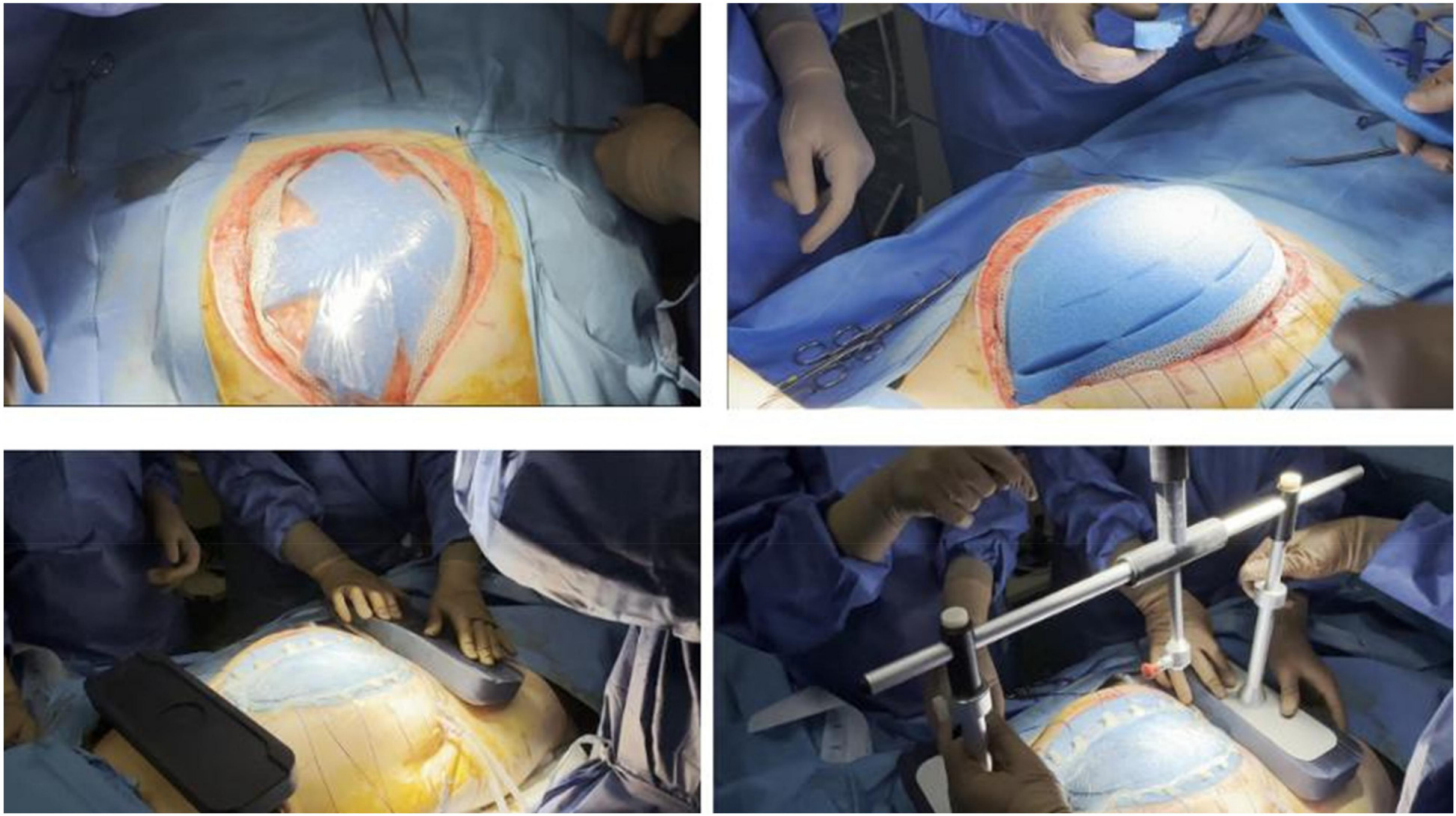
**(a–d)** Intraoperative application of the fasciotens® abdomen system. **(a)** Placement of titanised polypropylene mesh (TiMESH®) anchored to the fascial edges. **(b)** Sealing of the abdominal cavity with negative pressure wound therapy (Abthera®). **(c)** Positioning of tensioning sutures across the fascial edges. **(d)** Application of the fasciotens® Abdomen device with vertical dynamic traction.

Prior to device application, patients were placed under full intraoperative neuromuscular blockade to facilitate accurate measurement of the fascia-to-fascia (FTF) distance. Following abdominal lavage and surgical debridement to ensure clean and mobile fascial edges, a titanised type 1a polypropylene (TiMESH®, PMF Medical UK) was anchored to each fascial margin (Figure 1a). This macroporous mesh (pore size 1 mm), lightweight (16, 35, or 65 g/m^2^), monofilament, non-absorbable, and ethylene oxide-sterilised, featured laser-cut atraumatic edges. Titanium coating was selected to enhance tensile strength distribution and minimise the risk of fascial tearing because of its dense weave (16).

**Figure 2:**
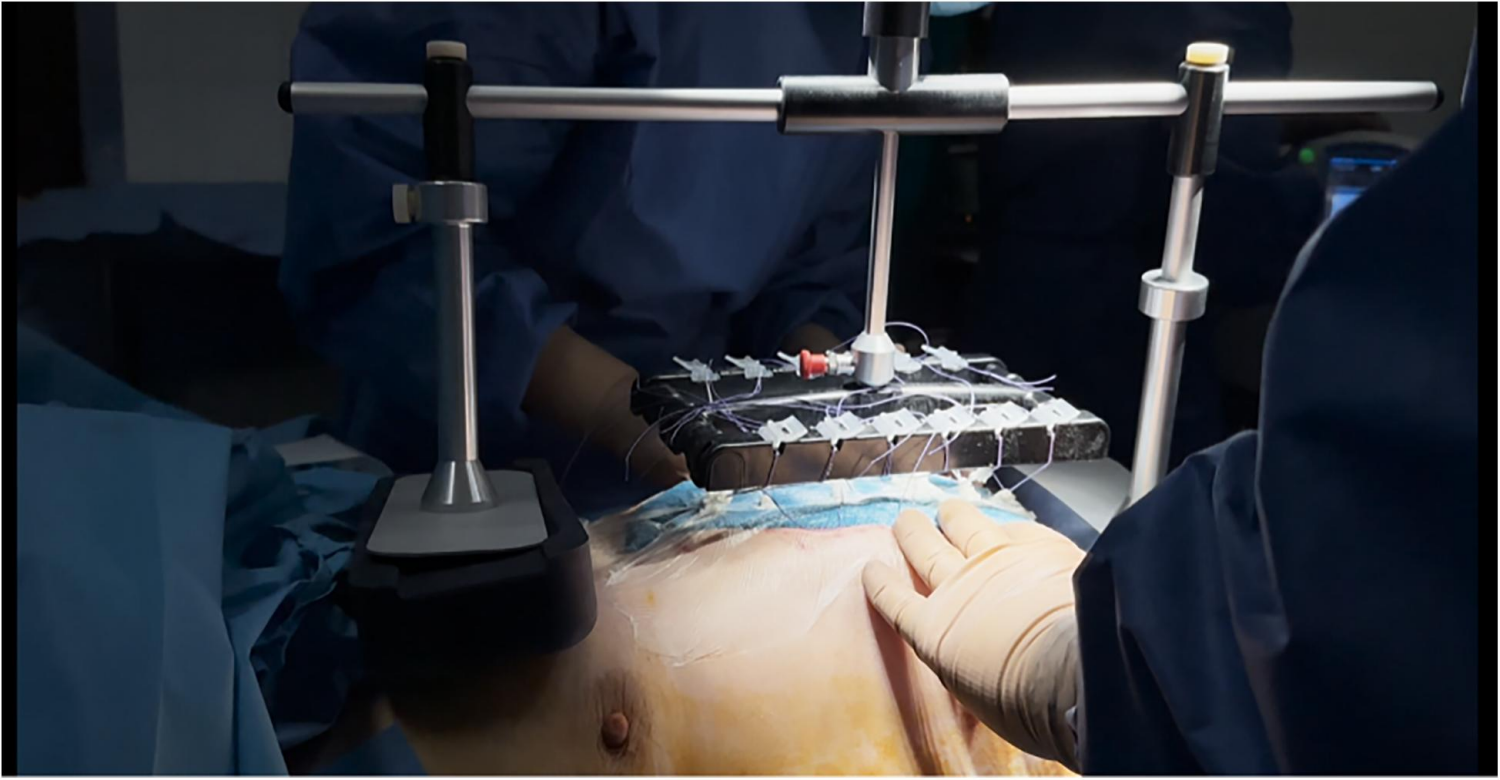
Final intraoperative setup of the fasciotens® abdomen system. The titanised polypropylene meshes are anchored to the fascial edges and connected via surgical sutures to the dynamic traction platform. The device applies continuous vertical traction (7–8 N) to approximate fascial margins while preserving tissue viability, with integrated adjustment mechanisms ensuring reproducible and quantifiable tension.

Subsequently, twelve Vicryl 2 surgical threads (made of polyglactin 910, which is a synthetic, absorbable copolymer made from a blend of glycolide and lactide), six on each side, were affixed to designated mesh points and tensioned vertically or diagonally using the clamping mechanism of the fasciotens® Abdomen device (Fasciotens GmbH, Essen, Germany). Negative pressure wound therapy (Abthera®, KCI Medical, Inc.) was used to seal the abdominal cavity (Figure 1b). Mesh sutures were prepared for attachment (Figure 1c). Using the integrated clamping mechanism, sutures were secured, and controlled dynamic traction was applied (Figure 1d). The final configuration of the system with sutures tensioned under the traction frame is shown in Figure 2. During subsequent abdominal explorations, performed 48–72 h after initial device application, the FTF distance was re-measured under complete patient relaxation.

Full neuromuscular blockade was applied exclusively during the index operation to facilitate accurate measurement of the fascia-to-fascia distance and secure mesh fixation under optimal conditions. Prolonged use of neuromuscular blocking agents in critically ill patients is associated with significant adverse effects, including critical illness myopathy or neuropathy, delayed ventilator weaning, respiratory complications, and disuse atrophy (17). For this reason, during subsequent traction adjustments and dressing changes in the ICU, no muscle relaxants were administered. This strategy was adopted to minimize the well-documented risks of prolonged neuromuscular blockade, particularly critical illness myopathy and delayed ventilator weaning, which can significantly impair patient recovery and functional outcomes. In our cohort, no cases of clinically significant ICU-acquired weakness attributable to the technique were observed.

To preserve fascial integrity, the doubled meshes were retained *in situ* as long as they remained functional, with replacement only when defects compromised traction. While in the ICU, traction force was assessed every 30–60 min using the traction head screw which helps maintain consistent tension during the procedure and adjusted as necessary to maintain consistent dynamic tension on the fascia. To minimize the risk of local pressure-related complications and to allow regular inspection of the fascial edges, the traction device was transiently released at predetermined intervals (approximately every 5 h) under sterile conditions in the ICU. These brief interruptions permitted direct assessment of suture integrity and fascial edge viability, after which the system was promptly reapplied and dynamic traction re-established. This protocol ensured continuous fascial approximation while preventing sustained pressure on any single area.

The pressure was maintained at 7–8 Newtons. Vertical traction was employed in cases of ACS or when primary closure was not feasible. Once edema subsided and visceral protrusion diminished, diagonal traction was applied to achieve myofascial elongation via lateral abdominal wall stretching, thereby facilitating fascial approximation and closure (18). The integrated device scale enabled quantifiable and reproducible traction adjustments tailored to individual anatomy.

IAP was monitored via intravesical catheter before and after device application, and at each subsequent revision, to assess abdominal compliance and compartment risk. Additional clinical parameters—including urine output, ventilatory indices, and vasopressor requirements—were systematically recorded as part of routine ICU care.

## 3 Case series- our results

### 3.1 Patient demographics

In our clinic, the vertical traction device was used in 13 patients over the past 24 months, including 5 women and 8 men, with a mean age of 58,8 years and a median age was 60.5. In 8 of the 13 patients, the indication for an open abdomen was peritonitis, in 3 cases intra-abdominal hemorrhage, in 2 cases ACS and in 1 case bowel obstruction due to generalized carcinomatosis. The etiologies of peritonitis in our case series included anastomotic leakage, such as leakage at the suture line of a small bowel anastomosis following prior surgery for intestinal obstruction (enterotomy for bezoar). Additional causes were peritonitis secondary to stoma retraction after total colectomy with end stoma formation and anastomotic leakage following intestinal repair in the context of trauma (gunshot injury). Baseline demographic and clinical characteristics of the study population are summarized in Table 1, which also allows for direct comparison between the hernia and non-hernia subgroups and provides essential context for interpreting the study results.

**Table 1:** Baseline demographic and clinical characteristics of the study cohort. Data are presented as mean ± standard deviation (SD), median (interquartile range, IQR), or number of patients (n) with percentages, as appropriate. The table also compares patients according to the presence or absence of hernia to facilitate subgroup analysis and contextual interpretation of outcomes.

Gender	*n* = 13	%	Non-hernia subgroup *n* = 8	%	Hernia subgroup *n* = 5	%
Male	8	61.54	5	62.5	3	60
Female	5	38.46	3	37.5	2	40
Age (years, median)	60.45				65.8	
Etiology for OA						
Peritonitis	7	53.85	4	50	3	60
Imminent ACS	2	15.38	–		2	40
Intra-abdominal hemorrhage	3	23.08	3	37.5	–	
Intestinal obstruction due to generalized carcinomatosis	1	7.69	1	12.5	–	
ASA						
III	3	23.08	2	25	1	20
IV	9	69.23	5	62.5	4	80
V	1	7.69	1	12.5	–	
Previous Abdominal Surgery	8	61.54	3	37.5	5	100%
Comorbidities						
Diabetes mellitus	4	30.77	3	37,5	1	20
Myocardial disease	6	46.15	6	75	–	
Arterial Hypertension	7	53.85	5	62,5	2	40
COPD	4	30.77	4	50	–	
Renal insufficiency	3	23.08	3	37.5	–	
Dyslipidemia	1	12.5	–		1	20
IBD (Ulcerative colitis, Crohn’s)	2	15.38	1	12.5	1	20
Osteoporosis	1	12.5	–		1	20
BMI						
18.5–24.9	2	15.38	1	12.5	1	20
25–29.9	9	69.23	6	75	3	60
30 or greater	2	15.38	1	12.5	1	20
ICU Treatment (prior to first surgery)						
Ventilation	8	61.54	7	87.5	1	20
Antibiotics	13	100	8	100	5	100
Vasopressors	8	61.54	6	75	2	40

OA, open abdomen; ACS, abdominal compartment syndrome; ASA, American society of anesthesiologist score; COPD, chronic obstructive pulmonary disease; IBD, inflammatory bowel disease; ICU, intensive care unit.

### 3.2 Indications for open abdomen and hernia subgroup

To account for the added complexity of patients with prior abdominal wall pathology, we divided the cohort into hernia and non-hernia subgroups. Patients with pre-existing hernias typically present with altered anatomy, prior mesh placement, or stoma formation, factors that increase the risk of postoperative complications such as sepsis and abdominal compartment syndrome. This subdivision allows for a more accurate assessment of outcomes and highlights the feasibility of traction-assisted fascial closure in this particularly high-risk group.

In our series, five patients had a pre-existing abdominal wall hernia at the time of their initial surgical intervention. All five developed major postoperative complications necessitating the use of the vertical fascial traction device for OA management. In all cases, the vertical fascial traction device was applied during OA treatment, demonstrating feasibility even in patients with a complex abdominal wall history. These cases underline the increased risk of postoperative complications in patients with hernia-related surgeries, and highlight the potential utility of traction-assisted fascial closure in this high-risk subgroup. This subgroup analysis can be found in Table 2.

**Table 2:** Subgroup analysis of patients with pre-existing abdominal hernia.

Sex	Initial surgery	Hernia context	Complication leading to OA	OA indication	Mesh usage	Fascial closure	Initial defect	Final defect
Male	Right colectomy with ileotransverse anastomosis	Incarcerated hernia → bowel resection	Anastomotic leakage from ileotransverse anastomosis after stoma closure	Fecal peritonitis	Death prior to definite closure	No	23 cm	Not applicable
Female	Ventral hernia repaired with Rives-Stoppa technique	Ventral hernia and cholecystectomy	Transverse colon rupture	Fecal peritonitis	Biological mesh-mediated closure	Yes	16 cm	10 cm (bridging)
Female	*ΤΜΕ* for rectal cancer	Large midline incisional hernia	Abdominal compartment syndrome due to bowel obstruction and diffuse peritoneal carcinomatosis	Abdominal compartment syndrome	Biological mesh-mediated closure (bridging)	Yes	14 cm	4 cm
Male	Right colectomy with ileotransverse anastomosis	Incarcerated hernia → bowel resection	Abdominal compartment syndrome	Abdominal compartment syndrome	No	Yes	18 cm	None
Male	Exploratory laparotomy- drainage placement		Perionitis due to colon rupture	Peritonitis	No	Yes	13 cm	None

In our study, we divided the cohort into hernia and non-hernia subgroups to acknowledge the distinct clinical and surgical challenges posed by patients with a pre-existing abdominal wall hernia. These patients frequently have a more complex abdominal wall anatomy due to prior surgeries, mesh placements, or stoma formations, which elevates their risk for postoperative complications such as abdominal sepsis and abdominal compartment syndrome. The feasibility and effectiveness of the vertical fascial traction system in facilitating open abdomen management can differ in this subgroup compared to those without hernias. By separately analyzing these patients, we provide valuable clinical insight into the system's performance in a high-risk population with compromised abdominal wall integrity. This distinction aids in patient stratification, risk assessment, and tailoring treatment strategies, ultimately supporting personalized and optimized management of open abdomen cases.

Within the hernia subgroup, five patients were treated with the vertical traction device. The first patient presented with a hernial defect measuring 23 cm and died before fascial closure could be achieved. The second patient had a 16 cm defect, which, after treatment, resulted in a residual 6 cm gap that was repaired with a bridging configuration. The third patient had a 14 cm defect and was left with a 4 cm residual gap, also managed with bridging mesh. In the fourth patient, the hernial defect measured 18 cm; primary fascial closure was achieved with the application of a prophylactic onlay mesh, without bridging. The fifth patient had a 13 cm defect and underwent successful primary fascial closure without the need for mesh bridging.

The decision to perform abdominal closure with bridging in selected patients was made to avoid component separation and thereby preserve reconstructive options for future surgery, in line with the recommendations of the European Hernia Society guidelines (19). In patients with a pre-existing hernia where primary fascial closure could not be achieved, bridging mesh repair was performed rather than component separation. Component separation was deliberately avoided in these cases to maintain future reconstructive options, particularly given the contaminated surgical field and the overall critical condition of these patients. Under such circumstances, component separation was considered less favorable due to its higher risk of wound morbidity and the disruption of native tissue planes, which would complicate any subsequent definitive abdominal wall reconstruction (20). The use of bridging mesh provided temporary closure of the abdominal wall defect while preserving the potential for a planned, more complex reconstructive procedure once the patient's condition improved and the surgical field was optimized. In our series, bridging was performed with Phasix® Mesh (BD, Becton, Dickinson and Company, Franklin Lakes, NJ, USA), despite its relative contraindication in non-sterile environments, based on intraoperative judgment of contamination risk and the clinical need for a biologically resorbable option in this critically ill cohort.

### 3.3 Device use and technical application

As part of the damage control surgery approach, during the initial surgery, no system was applied in order to keep the operative time short. In subsequent operations, there was an inability to close the abdominal wall, with an average fascial gap of approximately 16.3 cm, leading to the decision to apply the vertical fascial traction system. At the second-look laparotomy 48–72 h after the device was implemented, the average fascia-to-fascia (FTF) distance significantly reduced to 11.8 cm +/− 2 cm.

### 3.4 Outcomes

In our case series of 13 patients managed with open abdomen (OA), definite fascial closure (DFC) was achieved in 11 patients (84,62%), while 2 patients (15,38%) died prior to closure. In 9 out of 11 patients DFC was achieved with primary midline suture. From the 8 patients without pre-existing hernia, 7 underwent DFC and 1 patient died prior to closure. Among the 5 patients with pre-existing midline hernias, 4 achieved abdominal closure and 1 died before closure. Among the four patients in whom abdominal closure was achieved, two underwent direct fascial closure, while in the remaining two cases, closure was accomplished using a bridging mesh.

To further characterize the complexity of our cohort, patients were categorized according to the Björk Amended Classification (Table 3) (21). This classification system provides a standardized framework for assessing the severity of open abdomen cases. Notably, in patients classified as ‘frozen abdomen,’ the application of NPWT was particularly beneficial, as it compartmentalized the peritoneum from other intra-abdominal organs, limited dense adhesions, and facilitated safer surgical management.

**Table 3:** Bjork classification of the open abdomen.

Bjork amended classification	Number of patients	Percentage %
1a	1	7.69
1b	1	7.69
2a	3	23.08
2b	4	30.77
3a	2	15.38
3b	2	15.38

(1) Minimal complications with straightforward management; (1a) minor complications with no major abdominal issues, (1b) more significant complications (e.g., minor bowel involvement), (2) higher complexity with extensive abdominal or bowel involvement; (2a) Greater tissue compromise, (2b) extensive abdominal trauma. (3) severe complications requiring complex surgical interventions; (3a) major issues like significant bowel injury, (3b) critical cases with severely compromised abdominal contents.

The average number of revisions required until DFC was 2,53. The mean duration from the initiation of the traction device to fascial closure was 9,1 days in the general cohort (11/13 DFC), compared to 9,25 days in the hernia subgroup. The average fascia-to-fascia distance (FTF) at the time of OA was 16.3 cm, while in the hernia subgroup it was 17.8 cm. In line with findings from Eickhoff et al. (13) where a porcine model was used, a significant reduction in the initial FTF distance was observed between 48 and 72 h after device application.

Regarding the method of closure, mesh-mediated fascial closure was performed in 2 patients (18,18%), including both patients in the hernia subgroup. The remaining 9 patients (81,82%) underwent primary midline fascial closure. Negative pressure wound therapy (NPWT) was applied in all cases (100%).

Table 4 details the clinical course and outcomes of patients with open abdomen, including rates of definite fascial closure, number of surgical revisions, time to closure, and closure techniques used. Presenting these data highlights the effectiveness of NPWT and different closure methods in both hernia and non-hernia subgroups, providing critical insight into patient recovery and treatment efficacy.

**Table 4:** Open abdomen course.

Total	*n* = 13	%	Non-hernia subgroup *n* = 8	%	Hernia subgroup *n* = 5	%
Death prior to fascial closure	2	15.38	1	50	1	50
Definite fascial closure	11	84.62	7	87,.5	4	80
Total	*n* = 11		*n* = 7		*n* = 4	
Number of Revisions (average)	2.6		2.7		2.5	
Days until DFC	9.18		9.14		9.25	–
FTF at OA (cm)	16.2		16.1		16.3	
DFC method						
Mesh-mediated closure	2	18.18	0	0	2	50
Primary midline suture	9	81.82	7	100	2	50
NPWT	11	100	7	100	4	100

DFC, definite fascial closure; FTF, fascia-to-fascia distance; NPWT, negative pressure wound therapy.

Patients underwent an average 2,53 of dressing changes, and the system was used for approximately 8,9 days on average. Our patient outcomes can be found in Table 5.

**Table 5:** Our patient outcomes.

Patient	Device application Timing	Device duration (days)	Dressing changes	Closure type	Mesh use	APACHE	Charlson
1	3 days post-op	6	1	No closure (death)	No	20	4
2	6 days post-op	12	4	Primary closure	No	7	5
3	4 days post-op	9	3	Bridging with biosynthetic mesh (4 cm)	Bridging mesh	11	8
4	2 days post-op	6	2	Primary closure	No	19	
5	3 days post-op	9	3	Primary closure	No	11	1
6	6 days post-op	3	1	Primary closure	No	12	0
7	20 days post-op	15	4	Primary closure	No	9	1
8	Immediately post-op	9	3	No closure (death)	No	30	3
9	Immediately post-op	4	1	Primary closure	No	38	4
10	3 days post-op	20	5	Bridging with biosynthetic mesh (10 cm)	Bridging mesh	5	3
11	3 days post-op	6	2	Primary closure + prophylactic onlay mesh	Prophylactic mesh	15	3
12	6 days post-op	15	4	Primary closure + prophylactic onlay mesh	Prophylactic mesh	10	6
13	2 days post-op	2	–	Primary closure	No	35	2

APACHE, APACHE II score for the calculation of ICU mortality; Charlson, Charlson comorbidity Index for the prediction of one year mortality of hospitalized patients.

**Figure 3:**
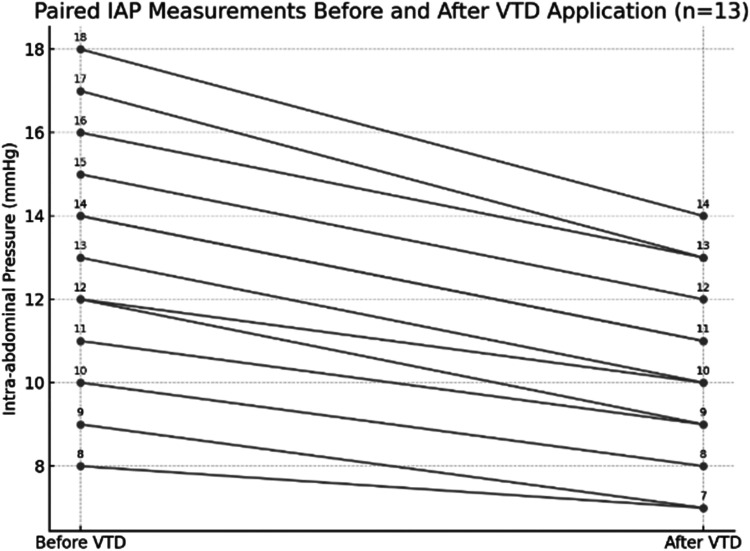
Paired intra-abdominal pressure (IAP) measurements before and after application of the fasciotens® abdomen in 13 patients. Each line represents an individual patient, demonstrating a consistent trend toward reduced IAP following device application.

**Figure 4:**
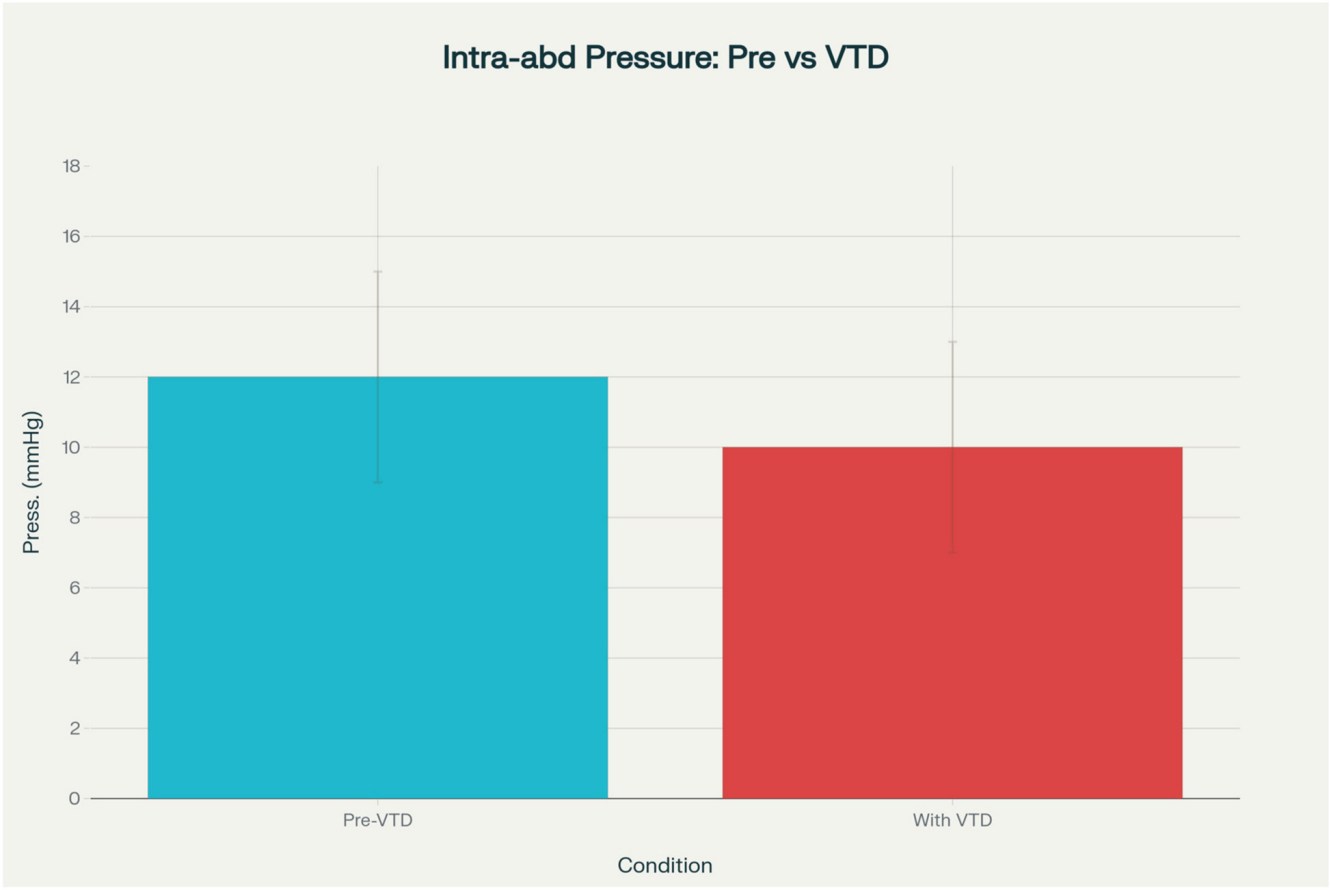
Intra-abdominal pressure prior and after application of VTD.

Intra-abdominal pressure (IAP) was systematically measured in all 13 patients prior to and following VTD placement. The mean IAP decreased from 12.8 ± 3.2 mmHg before device application to 10.1 ± 2.7 mmHg afterward (*p* = 0.06), suggesting a consistent trend toward improved abdominal compliance. Individual patient values and relevant paired trends in intra-abdominal pressure before and after application of the system are illustrated in Figure 3. No patient developed clinically relevant abdominal compartment syndrome after VTD application. Surrogate clinical markers, including preserved urine output, stable ventilatory parameters, and the absence of escalating vasopressor requirements, further supported the safety of the device. This finding indicates that the VTD does not increase intra-abdominal pressure during open abdomen management and may contribute to modest decompression in certain cases, thereby supporting its safety in maintaining physiologic IAP levels. The bar chart can be found in Figure 4.

In our case series, the vertical traction device (VTD) was applied in combination with a negative pressure wound therapy system in all patients and no temporary abdominal closure system was used.

### 3.5 Complications and follow-up

There were no complications directly related to the application of the VTD, and no cases of intra-abdominal abscesses, entero-atmospheric fistulas, or wound infections occurred while the device was in use. After successful fascial closure, none of the patients experienced wound dehiscence. During follow-up, no additional laparotomies were needed. However, one patient developed a superficial wound infection after definitive fascial closure (DFC), which was effectively managed with NPWT. This therapy was continued post-closure until the skin was fully healed. Additionally, no cases of fascial dehiscence were observed during hospitalization. During follow-up (mean 10 months, range 6–14 months), no cases of delayed entero-cutaneous fistula (ECF) were identified. Furthermore, none of the patients developed late wound dehiscence or required re-laparotomy after definitive closure.

## 4 Discussion

The term “open abdomen” describes a condition where the abdominal wall remains open, exposing the abdominal viscera. This approach is commonly used in damage control surgery for conditions such as sepsis, trauma, or abdominal compartment syndrome. Management involves temporary closure techniques.

The open abdomen approach is primarily indicated in clinical scenarios requiring damage control surgery (DCS), including severe trauma, abdominal compartment syndrome (ACS), peritonitis with extensive contamination, and certain cases of vascular emergencies, such as ruptured abdominal aortic aneurysms (1). The open abdomen technique allows for the control of hemorrhage, the mitigation of abdominal hypertension, and the staged management of severe infections (22–27). By maintaining the abdomen in an open state, further compromising the patient's physiology is avoided, enabling re-exploration as needed (28). and offering a potential survival benefit associated with the DCS approach (29).

This prospective series demonstrates that application of the Fasciotens® Abdomen (VTD) in open abdomen (OA) management is feasible, safe, and facilitates high rates of delayed primary fascial closure, including in patients with complex pre-existing hernias. Our cohort achieved a closure rate of 84.6%, which compares favorably with previously reported outcomes of traction-assisted closure techniques and exceeds those historically observed with NPWT alone, where closure rates of 60%–70% have typically been described (30). These findings highlight the added value of dynamic fascial traction in preventing progressive lateral retraction of the abdominal wall, thereby increasing the likelihood of primary closure.

Our DFC rate of 84.6% aligns with prior reports of traction-assisted closure techniques (31). Importantly, this study extends the evidence by including patients with pre-existing hernias, a subgroup rarely represented in published cohorts.

In our series, the most frequent indication for OA was abdominal sepsis (53.85%), followed by intra-abdominal hemorrhage (23.08%), abdominal compartment syndrome (15.38%), and intestinal obstruction due to carcinomatosis (7.69%). These findings align closely with the typical emergency indications described in the literature, confirming that our patient population reflects a representative spectrum of OA scenarios.

ACS itself is another common cause, occurring when intra-abdominal pressure rises due to excessive fluid accumulation within the confined abdominal cavity, often from resuscitation-related fluid overload, internal bleeding, or ascitic fluid buildup (32–34). Abdominal sepsis, typically caused by bowel perforation and leading to persistent intra-abdominal infections and abscesses, may also necessitate an open abdomen. Our patients with septic OA primarily suffered from anastomotic leaks or complications following stoma creation or closure, including cases following enterotomy for bezoar, trauma-induced small bowel injury, and complex colorectal anastomoses. In such cases, negative pressure wound therapy helps maintain abdominal domain, facilitates fluid drainage, allows for repeated irrigation, and reduces the risk of postoperative infections while improving fascial closure outcomes (35–37).

Leaving the abdomen open can be necessary in certain cases, but it also comes with complications such as fluid loss, bowel exposure, and muscle retraction. Significant fluid can escape from the abdominal cavity, which may lead to hypovolemia if not properly managed. Additionally, protein-rich peritoneal fluid is lost at a rate of about 2 grams per liter, making nutritional adjustments essential (38).

Another risk is fistula formation, particularly enterocutaneous or enteroatmospheric fistulas, which develop in up to 20 percent of the cases and can occur as early as eight days from the initial laparotomy (39, 40), especially in patients with bowel anastomoses. Furthermore, prolonged abdominal opening can cause muscle retraction, making it difficult to achieve primary closure later, leading to a large ventral hernia.

Using negative pressure wound therapy combined with the fascial traction system may counteract this effect, improving the chances of closing the fascia or at least minimizing the hernia size (41).

Several methods have been proposed for the repair of the open abdomen including Bogotá bag, Wittmann Patch®, skin closure only, dynamic fascial traction devices, and negative pressure wound therapy (NPWT) with or without the use of a fascial traction device (42–45). Negative pressure wound therapy with continuous fascial traction is suggested as the preferred technique for temporary abdominal closure (Grade of Recommendation 1B) (46). The vacuum-assisted wound closure and mesh-mediated fascial traction (VAWCM) technique has become the most established method for temporary abdominal closure when delayed primary fascial closure is anticipated. This approach combines negative pressure wound therapy with an interposed mesh sutured to the fascial edges, which is gradually tightened at subsequent revisions. VAWCM has been shown to improve delayed fascial closure rates compared with NPWT alone, while reducing mortality and the need for permanent ventral hernia (47). Nevertheless, the method is not without limitations. It requires repeated surgical re-explorations for mesh adjustment, closure rates vary between centers, and complications such as enteroatmospheric fistula or large incisional hernia can still occur (48). Despite these drawbacks, VAWCM remains the reference standard against which newer dynamic traction systems, such as fasciotens®, are compared.

The fasciotens® Abdomen system provides dynamic, quantifiable fascial traction that counteracts lateral retraction and promotes myofascial elongation, thereby facilitating delayed primary fascial closure (49). In contrast to conventional temporary abdominal closure techniques, it enables reproducible bedside adjustments, preserves tissue viability, and reduces the need for complex reconstructive procedures such as component separation. Clinical studies have reported higher rates of delayed fascial closure and reduced incidence of giant ventral hernia with the use of fascial traction devices (50).

In accordance with current recommendations, all patients in our cohort were treated using NPWT in combination with vertical fascial traction. Specifically, an AbThera™ sponge system was used in every case, which provided consistent negative pressure while allowing the traction device to function effectively. This uniform approach aligns with best practices for minimizing fascial retraction and promoting early closure (14).

Notably, in our 13-patient cohort, we observed no cases of entero-atmospheric fistula formation, despite a high proportion of patients with prior abdominal surgery and septic etiology. This supports the safety profile of NPWT when used in conjunction with vertical fascial traction and suggests that careful technique may mitigate even the baseline risk seen in large-scale meta-analyses.

The application of negative pressure opposes the lateral retraction of the abdominal musculature, minimizing loss of domain and improving the likelihood of primary fascial closure (51). Our data also reflect this benefit: in most cases, progressive reduction in fascia-to-fascia distance was observed during the first 48–72 h, ultimately leading to an 84.62% definitive fascial closure rate without the need for permanent mesh bridging. These findings are consistent with previous reports, where NPWT in combination with fascial traction has yielded closure rates of 70%–90% (14). This highlights the critical role of negative pressure therapy, particularly when paired with active vertical traction, in maintaining abdominal domain and avoiding the need for complex abdominal wall reconstructions.

### 4.1 Role and benefits of vertical fascial traction in OA management

Adjunctive fascial traction techniques can help bring the fascia toward the midline, improving the chances of primary closure (51–54). However, excessive manipulation or tension should be avoided to prevent fascial injury or intra-abdominal hypertension. Various methods are used, including vessel loops for skin traction, commercial closure systems like the Abdominal Re-approximation and Anchor System (ABRA) (44, 55), mesh-mediated tension such as the Wittman patch, and fascial tension sutures (9) or retention sutures.

A systematic review by the Eastern Association for the Surgery of Trauma (EAST) analyzed four trials comparing fascial traction (51, 56–58), with or without negative pressure, to negative pressure alone. The findings showed that using fascial traction significantly lowered the failure rate of primary fascial closure during the initial hospital stay (19.7% vs. 40.1%) and reduced the incidence of ventral hernias (9.8% vs. 45.4%) (53).

These findings are supported by our own experience, where vertical fascial traction enabled successful midline fascial closure, despite the high-risk nature of the cohort. Among the patients who achieved closure, no early ventral hernia or wound dehiscence was observed during follow-up. Additionally, fascial traction did not lead to an increased risk of enterocutaneous fistula formation or mortality.

### 4.2 Comparative effectiveness and outcomes of fascial traction methods

Mesh-mediated horizontal traction remains a commonly used technique for managing the open abdomen (OA), particularly in situations where delayed primary closure (DPC) is anticipated (15). This method involves placing a mesh material across the fascial defect to apply tension and gradually approximate the fascial edges. Even though mesh-mediated horizontal traction facilitates gradual fascial approximation and prevents the loss of domain (46), the horizontal tension applied by the mesh may exacerbate intra-abdominal hypertension, potentially leading to abdominal compartment syndrome (59).

The challenge posed by an open abdomen as a damage control measure lies in the retraction of the fascia from the midline, leading to a loss of intra-abdominal volume. To address this issue, the previously employed method of horizontal fascial tension was associated with several drawbacks. These included increased intra-abdominal pressure due to the protrusion of intra-abdominal viscera (59), the necessity for repeated fascial tensioning,. In contrast, the method of vertical fascial traction, which is applied immediately after laparotomy, prevents fascial retraction, reduces intra-abdominal pressure, and facilitates secondary closure of the abdomen.

### 4.3 Clinical experience with vertical traction device in this and prior studies

Additionally, a case report by Nguyen et al. discussed the use of a vertical traction device in combination with other therapies for open abdomen management. The authors suggested that vertical traction may be advantageous over horizontal traction, particularly in the presence of intestinal edema during the initial phase of open abdomen care. However, this report did not provide specific data on ACS rates (50).

More robust evidence was presented by Fung et al. in a multicenter study including 20 patients with both septic and non-septic open abdomen (60). The study evaluated the impact of a novel vertical traction device (VTD) on primary fascial closure (PFC) and prevention of fascial retraction. Their findings demonstrated that the combination of VTD with negative pressure wound therapy (NPWT) effectively prevented fascial retraction, promoted rapid fascial closure of large abdominal defects, and reduced the need for complex abdominal wall reconstruction or mesh grafting. The direct fascial closure rate in their cohort was 100%.

In our series, a definite fascial closure was achieved in 84.6% of patients overall, which, while slightly lower than the 100% closure rate reported by Fung et al., still supports the effectiveness of the vertical traction device even in a heterogeneous, high-risk population (ASA III–V in 91.6% of cases). Notably, when excluding patients with pre-existing hernias, the fascial closure rate reached 100% (7/7), underscoring the efficacy of the device in primary open abdomen management. Our median time to fascial closure was 9.1 days overall and 9.25 days in the hernia subgroup, consistent with the timeframe reported by Fung et al. (7–7.5 days).

Regarding fascia-to-fascia (FTF) distance, Fung et al. reported an average of 17.5 cm in the VTD–NPWT group, which decreased to 14 cm after 48 h, compared with a reduction from 13 cm to 9.5 cm in the VTD–TAC group. In our cohort, the average initial FTF distance was 16.3 cm (range: 16–19 cm), with a consistent reduction to 10 cm within 48 h of traction. These comparable findings further support the reproducibility of the technique across different patient populations.

### 4.4 Timing of closure and impact on morbidity and mortality

Another study compared early fascial closure with delayed abdominal closure in OA management. The results demonstrated that early fascial closure significantly reduced mortality and complication incidence compared to delayed closure. The mean interval from OA to definitive closure ranged from 2.2 to 14.6 days in early closure groups, highlighting the benefits of timely intervention in reducing adverse outcomes (8). Furthermore, research assessing a novel VTD designed to facilitate early fascial closure in OA patients reported that the device was effective in preventing significant complications associated with OA therapy, such as abdominal infections, entero-atmospheric fistula (EAF), and abdominal wall hernia formation. The study concluded that early definitive fascial closure is crucial in OA management, and the use of VTDs can play a significant role in achieving this goal (61).

### 4.5 Special considerations in patients with pre-existing hernias

In our study, five patients had a pre-existing abdominal wall hernia and underwent complex procedures, including mesh placement or stoma closure, prior to OA. Despite increased technical difficulty, four out of five achieved definitive closure with the aid of the vertical traction device. These results support the feasibility of this technique even in challenging subgroups.

Further case reports, such as one by Mavc and Kunz, highlighted the added advantages of using a vertical fascial traction device, such as a swift enhancement in the patient's respiratory function, increased urine output, improved stoma output, and better stabilization of hemodynamic parameters (62). Nevertheless, the absence of a control group and the lack of long-term follow-up underline the need for further research.

### 4.6 Post-closure monitoring, fluid management, and resuscitation strategies

After temporary abdominal closure, patients are typically monitored in the intensive care unit (ICU), where dressings, such as adhesive, gauze, or negative pressure systems, are changed as needed, and the abdominal contents are inspected every two to three days, either in the ICU or the operating room, depending on the patient's condition and the reason for keeping the abdomen open. Excessive fluid administration, particularly with crystalloids, can worsen visceral edema and hinder fascial closure, while alternative resuscitation strategies, such as using colloid or hypertonic solutions, may help reduce interstitial swelling and promote closure (63, 64).

Some studies suggest that direct peritoneal resuscitation (DPR) with hypertonic peritoneal dialysate fluid can improve splanchnic and hepatic blood flow, decrease bowel edema, and enhance the chances of fascial closure, although there is no standardized protocol for its use (65, 66). While many patients with an open abdomen remain intubated due to their critical condition, mechanical ventilation is not always required (67).

### 4.7 Definitive closure techniques: primary and functional closure

Once the indication for the open abdomen has resolved, the abdomen is closed. The preferred approach for closing an open abdomen is primary fascial closure, in which the edges of the fascia are directly approximated, as it results in the lowest complication rates. In our study, primary fascial closure was achieved in 81.82% of the patients using direct midline suturing. The remaining 18.18% required biologic mesh-mediated closure, reflecting both the feasibility and limitations of achieving primary closure in a mixed-diagnosis cohort. We acknowledge that the use of permanent or bridging mesh in the context of an open abdomen is controversial, as TAC inherently implies a contaminated wound. In our series, mesh was reserved for patients with pre-existing hernias where direct fascial closure was not achievable. The decision was individualized, balancing the risks of mesh placement in a contaminated field against the need to avoid complex component separation and preserve options for delayed definitive reconstruction.

However, ventral hernias can still develop in up to 30 percent of cases following this method (7). To mitigate this risk, biologic mesh reinforcement may be used, typically as an underlay, though an inlay or bridging technique may be necessary if the fascia cannot be approximated. Additionally, component separation techniques can facilitate fascial apposition when necessary (68–70).

If primary closure is not possible, functional closure can be performed using biologic mesh as a bridge over the fascial defect. This mesh acts as a scaffold for host cell repopulation, promoting the formation of new fascial tissue (11–13). The skin is then closed over surgical drains placed in the subcutaneous space. However, functional closure is not recommended if the skin cannot be approximated over the biologic mesh, as exposed mesh is prone to degradation and infection, a process that can take weeks to resolve.

The long-term risk of ventral hernia after functional closure remains uncertain, though one study reported an 80% hernia rate at an average follow-up of 21.4 months in patients who underwent ventral hernia repair with acellular dermal matrix (71). Another review of 37 patients found that closure using human acellular dermal matrix was feasible in all cases of open abdomen due to damage control surgery, with early closure associated with fewer complications, although “early” was not explicitly defined in the study, where the mean open abdomen duration was 21 days (72). By contrast, our average OA duration post-device application was 8,9 days, with only two patients requiring biologic mesh bridging. While our follow-up data are limited, none of the patients with successful closure developed early mesh-related complications, suggesting that earlier intervention with vertical fascial traction may reduce the need for functional bridging and its associated long-term risks.

In our study, pre-existing abdominal wall hernia was a significant factor contributing to the complexity of the clinical course in this subgroup, often necessitating high-risk surgical interventions and increasing the likelihood of requiring open abdomen management. All five patients underwent staged procedures, such as protective ileostomies or fascial closure with mesh, which were associated with delayed complications, including anastomotic leakage and abdominal compartment syndrome. Notably, all complications arose at or near anastomotic sites, underscoring their vulnerability in the context of hernia-related surgeries. Despite these complexities, the vertical fascial traction device was successfully utilized in all cases, supporting its feasibility even in critically ill patients with a challenging abdominal wall, including those with prior mesh placement.

Our findings suggest that surgeons should anticipate postoperative complications in patients with a history of hernia surgery, particularly when stomas or prosthetic materials are involved. Early consideration of traction-assisted closure techniques may enhance the likelihood of successful fascial approximation and potentially reduce the need for permanent stomas or result in smaller ventral hernias. However, long-term outcomes, such as definitive fascial closure rates and hernia recurrence, remain essential metrics to further validate the efficacy of this technique in this high-risk subgroup.

### 4.8 Management of planned ventral hernia

If neither primary nor functional closure is possible, a planned ventral hernia remains the only option. This can be managed by either skin-only closure, which involves approximating the skin over the fascial defect while leaving a hernia, or by applying a split-thickness skin graft if the skin cannot be brought together. In cases where skin approximation is not feasible, the abdominal contents are allowed to adhere to each other and the abdominal wall until a layer of granulation tissue forms over the bowel, at which point a skin graft can be applied (73). Sometimes, an absorbable mesh, such as Vicryl, is temporarily sutured to the skin to prevent evisceration while waiting for granulation. Before elective hernia repair is attempted, a waiting period of six to twelve months is generally recommended to allow for adhesion maturation and decrease the risk of bowel injury during surgery (74).

### 4.9 Review of outcomes and risk factors for failed fascial closure

Results from systematic reviews and studies indicate that the success of primary fascial closure varies depending on the technique used. One review analyzing 74 studies reported fascial closure rates ranging from 34 to 74 percent, with fistula formation occurring in 2.2 to 29.5 percent of cases and mortality rates between 11 and 39 percent among patients requiring open abdominal management (48).

Risk factors for failed primary closure in a study of 572 patients included multiple re-explorations, intra-abdominal sepsis, enteric fistula, and an Injury Severity Score (ISS) above 15 (75). In non-trauma patients, peritonitis or the presence of a stoma also increased the likelihood of closure failure (76).

Comparative studies have shown that the Wittmann Patch achieves the highest primary fascial closure rates at approximately 90 percent (4 studies/180 patients) (77), with additional studies reporting closure rates of 78 and 82 percent (43, 78).

Some studies have reported higher closure rates for negative pressure systems, ranging from 22 to 91 percent, reflecting variability in study populations and methodologies (48, 79). A retrospective review of 104 patients found that negative pressure systems significantly improved closure rates compared to cases without abdominal tension (78 vs. 44 percent) (52). Additionally, combining fascial traction with negative pressure therapy further improved closure rates to 73 percent (range 63 to 81 percent) compared to 52 percent (range 47 to 56 percent) with negative pressure therapy alone (48).

### 4.10 Strengths and limitations

This study provides real-world data on the use of a vertical fascial traction device for open abdomen management in a high-risk, heterogeneous patient cohort, including individuals with abdominal sepsis, hemorrhage, and compartment syndrome. One of the main strengths of our study is the consistent use of a standardized protocol, including early application of vertical traction and the Abthera negative pressure system, which allows for uniform evaluation of outcomes. Additionally, our findings contribute valuable clinical insight into the device's feasibility in complex scenarios, such as prior mesh placement, staged stoma formation, and hernia-related surgeries—contexts often underrepresented in the literature.

The relatively small sample size is an inherent limitation of this study and restricts the statistical power of our findings. However, given the rarity of open abdomen cases requiring vertical fascial traction, even small series contribute important preliminary data. Notably, our inclusion of patients with pre-existing abdominal wall hernias—an underrepresented subgroup in the literature—provides novel insights despite the limited cohort size. The lack of a contemporaneous control group treated with alternative closure techniques also precludes direct comparison of efficacy. Furthermore, long-term follow-up data on incisional hernia development rate or functional abdominal wall outcomes were not available for all patients.

Despite these limitations, our study highlights the potential of vertical traction as a practical adjunct in the multidisciplinary management of the open abdomen.

This study's limitations also include its design as a single-center prospective cohort which may limit the broader applicability of the findings. While small cohort studies are essential for initial evaluations of new surgical techniques and infrequent clinical indications, they inherently lack the diversity and scale offered by multicenter collaborations.

Emergency abdominal surgery poses challenges for traditional randomized controlled trials (RCTs), making multicenter registries and international cooperative databases particularly valuable. Such registries facilitate larger patient inclusion, reduce selection bias, and capture the heterogeneity of patient populations and surgical practices, improving generalizability and evidence strength.

Future investigations would benefit from integrating this technique into broader multicentric registries or cooperative studies to validate and refine its clinical utility across diverse settings. Meanwhile, our single-center data provide important preliminary evidence supporting the safety and effectiveness of vertical fascial traction combined with NPWT in complex open abdomen management.

## 5 Conclusions

The method of vertical fascial traction represents a pivotal improvement in the management of the open abdomen. Its application addresses the challenges of fascial retraction and elevated intra-abdominal pressure, promoting effective delayed closure and improving overall patient outcomes. Further research and refinement of this technique may continue to enhance its utility in clinical practice. Nevertheless, our experience, congruent with existing literature, suggest positive outcomes in the management of patients with open abdomen, facilitating the closure of the open abdomen. Collectively, our experience highlights the vertical fascial traction device as a safe, effective, and technically straightforward alternative to traditional horizontal mesh-mediated techniques, offering promising outcomes in terms of closure rates and complication avoidance, even in complex and critically ill surgical patients.

## Data Availability

The original contributions presented in the study are included in the article/Supplementary Material, further inquiries can be directed to the corresponding author.
